# Supplementary material for: Are carbon emissions trading and green financial instruments synergistic? -Comprehensive quantitative research based on content analysis
Source: PLoS One. 2024 Mar 7;19(3):e0298601. doi: 10.1371/journal.pone.0298601 (PMC10919601; doi:10.1371/journal.pone.0298601)
Supplement: S1 Data — (ZIP) [file pone.0298601.s001.zip › Data/地级市Z_F工作报告.zip!/║┌┴·╜¡/║╫╕┌╩╨/└┤╫╘╣1⁄2╓┌║┼í╛┬φ┐╦╩2╛▌═°í┐_2016.docx]

各位代表：

　　现在，我代表市人民政府，向大会报告工作，请予审议。并请各位政协委员和其他列席人员提出意见。

　　一、“十二五”时期简要回顾及2015年工作情况

　　“十二五”时期，是鹤岗发展史上非同寻常、极不平凡的五年。特别是近三年，经济社会发展进入最为艰难的时期,在承受空前经济下行压力的同时，更经历了超百年一遇的特大洪水、50年一遇的雪灾以及多起矿难和森林火灾。我们在省委、省政府和市委的坚强领导下，在市人大、市政协的支持与监督下，团结和带领全市人民主动适应经济发展新常态，沉着应对困难和挑战，科学完善发展思路，妥善处理矛盾和问题，心无旁骛抓解困，凝心聚力谋发展，呈现出经济发展止跌向好的态势，发展后劲明显增强、民生持续改善、社会安定和谐、各项事业稳步发展。地区生产总值由2013年下降9.5%、2014年下降9.7%，到2015年增长4%；规模以上工业增加值由2013年下降28.4%、2014年下降39.5%，到2015年增长5.3%；固定资产投资累计实现701.6亿元，是“十一五”时期的1.6倍；公共财政收入由2013年下降24.5%、2014年下降18%，到2015年下降7.3%，降幅持续收窄17.2个百分点；社会消费品零售总额实现116亿元，年均增长10.2%；外贸进出口总值2.2亿美元，年均增长21.9%；城镇居民人均可支配收入19400元，年均增长10%；农村居民人均可支配收入12610元，年均增长7.3%，比2010年翻一番。项目建设不断加快，实施千万元以上产业项目370个，其中亿元以上项目154个，产业项目投资占固定资产投资的比一直保持在65%以上。经济结构不断优化，非煤产业比重由2010年的26.3%调整到40.1%。城乡面貌不断改善，实施了沉陷区治理和棚户区改造，以及路网、水网、热网、气网、电网等一系列城市重大基础设施工程，既有建筑节能改造、小城镇建设、农村泥草房改造等城乡建设重点工作顺利推进。民生事业不断进步，坚持越是困难时期越是关注民生的理念，累计民生投入超过292亿元，是“十一五”时期的2.6倍。就业规模持续扩大，社会保障体系、住房保障制度逐步完善，教育、卫生、文化、体育、科技等事业全面发展。改革开放不断深入，政府机构改革、事业单位分类改革、农村综合改革和医疗卫生、教育、公共服务等领域改革取得积极成果。累计利用外资2.3亿美元，是“十一五”时期的3.2倍。可以说，过去的五年，是理思路、定战略、抓发展的五年，更是化危机、破难题、抓解困的五年，也是打基础、利长远、抓转型的五年。

　　各位代表！2015年是“十二五”的收官之年。面对主导产业市场持续低迷、主要产品量价齐降、融资瓶颈凸显、改善民生和维护稳定压力增大等多重困难交织叠加的严峻形势，市政府和全市人民一道，迎难而上，奋发有为，努力克服经济下行带来的不利影响，经济实现止跌回升，社会保持和谐稳定。

　　——我们坚持长远谋事，着力推进招商引资和项目建设，发展后劲逐步增强。始终把项目建设作为“经济增长、财税增收、就业创业”之源，围绕“煤墨绿贸游”五大产业，大上项目，全年共开复工千万元以上产业项目132个。聚力推进煤电化工产业，中海油华鹤大化肥、征楠煤化工LNG等一批重点项目建成投产，鹤翔新能源焦炉煤气制LNG项目开工建设，征楠煤化工二期等项目加快推进。强力推进高端石墨产业，高起点编制石墨产业专项规划，开展石墨探矿权、采矿权“两清、两整”行动，组建了鹤鸣云山矿业开发有限公司，增强了政府对资源配置、园区建设、合作开发和融资发展的掌控权，为石墨产业大发展、强发展、跳跃发展理清了思路，清除了障碍，奠定了基础。全力推进绿色食品产业，华润粮食加工、万源3万吨米糠毛油精细加工、吾朋生物科技大米蛋白粉深加工等项目建成投产，关东米业、兴汇山葡萄酒、利农万吨玉米深加工等项目开工建设，绿色食品产业实现销售收入60亿元。大力推进科技型新兴产业项目，积极实施“全省千户科技型企业三年行动计划”，新增科技型企业48户，无糖型安神宁口服液等5个项目列入省科技计划，经纬糖醇公司被认定为国家高新技术企业，秀泉科技氧化法清洁制浆、鹏程科技轨道交通吸音板、吉阳新能源光伏电站等项目开工建设。积极推进招商引资，通过参加国家、省组织的经贸活动和赴韩国、港澳台、环渤海、长三角、珠三角等地开展专项招商推介活动，共引进项目144个，总投资327亿元，到位资金80.8亿元。

　　——我们坚持科学干事，着力推进存量盘活和增量做大，整体经济止跌回升。按照“一产做精、二产做强、三产做活”的思路，将全年目标任务制成《责任指标体系分解监测总册》，盯死目标抓落实，全力以赴抓运行、保增长、促发展。农村经济稳步增长。粮食产量稳定在30亿斤，实现“十二连增”。各类新型农村经营主体达到447家。建设了3条百里现代农业示范带，21个农机化千亩科技示范园区，农业机械化率提高到98%。“三品”种植面积达到200万亩、标识300个。肉蛋奶总产量达到6.8万吨，畜牧水产业增加值增长5%。德龙、福兴灌区工程按计划推进，关门嘴子水库可研工作全面启动。投资1.2亿元，推进新农村建设和美丽乡村建设。整村推进12个村、1.7万人扶贫脱贫。工业经济逆势回升。出台了《支持地方工业发展若干政策措施》、《关于支持龙煤鹤矿脱困发展的实施意见》，完善了企业服务中心，建立了企业投诉中心，发放助保金2.7亿元。严格落实各级领导、各部门包企责任制，成立10个专项推进组，全面展开“双攻坚”行动，及时有效化解企业遇到的各类难题，确保存量增效、增量达效。新增规上企业13户，规模以上工业增加值增幅列全省第二位。在全国首创由地方政府组建的企业家学院，打造提升素质、沟通交流、合作发展的企业家教育服务平台，引起社会各界强烈反响。第三产业提档增速。“汽车4S店一条街”等10个大型商贸流通项目顺利实施，国际物流货运枢纽站项目已通过省发改委评审。组建了旅游集团总公司，龙湖生态旅游度假区、嘟噜河湿地景区、山水风景区、中国北方共青城、宝泉岭森林博物园5个景区被评为国家3A级旅游景区。金羽丝路创意产业基地和名山影视文化产业园列入省重点推进项目，六公里生活圈手机终端软件顺利进入市场。金融机构本外币各项存贷款余额565.8亿元和453.6亿元，增长16%和28%。赴俄锰铁矿项目有序推进，出产矿石4万吨；赴俄农业种植面积达到1.9万公顷，增长15%。

　　——我们坚持朴实做事，着力推进民生实事和社会管理，人民生活持续改善。秉承“民生为重、民生为先、民生为上”的理念，把保障和改善民生作为一切工作的出发点和落脚点，千方百计调动可用财力向民生投入、工作力量向民生倾斜，切实解决好涉及老百姓切身利益的热点、难点问题，让人民群众共享改革发展成果。民生工程深入实施。积极落实就业创业政策，建立大学生创业基地，为有创业愿望人员发放小额担保贷款、农村妇女创业贷款6161万元，支持大众创业、万众创新。全年实现城镇新增就业2.4万人，下岗失业人员再就业1.5万人，城镇登记失业率4%。继续实施“五险”扩面，新增参保人员2.9万人，企业离退休人员基本养老金由2014年的1540元/月提高到1756元/月。持续开展“寒冬送温暖”等救助活动，向社会困难群体发放救助金4.5亿元。建设社区日间照料中心43个，创建“双百”示范社区6个。大力推进各类保障性住房建设，启动了总投资41.5亿元、2.4万户的采煤沉陷区棚户区改造工程。投资20.7亿元，新建各类保障房20329套，结转续建3238套，购买存量商品房3000套。安置回迁5102户，货币补偿2000户，分配入住公租房3000套，改造农村泥草房771户。社会事业不断进步。各级各类教育协调发展，新创建省级标准化学校8所，对42所学校进行信息化建设，改建幼儿园6所。创建6所学生综合实践活动基地和1所青少年科技创新实践活动基地。鹤岗一中成为全国16所空军青少年航空学校之一。绥滨县被确定为全省第二批省部共建国家现代农村职业教育改革试验区试点县。鹤岗师专“专升本”成绩名列全省同类院校第一名。医疗卫生服务功能日趋完善，改造、新建了市精神病院附属楼和卫校农村医学综合楼，市妇幼保健院综合楼投入使用。人均基本公共卫生服务经费标准提高到40元，新农合人均筹资标准达到470元。实施医疗服务惠民工程，共减免医药费830万元。公立医院改革全面实施，萝北县国家级公立医院改革试点全省排名第一。大力发展养老服务业，市中医院“医养康”综合体、鹤矿集团兴安分院“医养康”服务中心项目快速实施。文化、体育事业蓬勃发展，建成了33个农村文化广场、54个社区文化活动中心和文化活动室，各类文化场馆全年免费接待13万人次。组织创作各类文艺作品1000余件，举办各类文艺演出66场。实施了广播电视户户通工程，开通了地面数字电视节目。广泛开展全民健身运动，加强群众身边体育设施建设，新建户外活动基地2个、各类健身工程126套。竞技体育水平不断提高，获得国家级以上比赛奖牌19枚。加强人才工作，认证市级领军人才126人。社会形势持续稳定。创新信访工作机制，全力破解信访难题，实现了信访总数、到省信访和进京非正常访“三下降”。大力推动安全责任体系建设，开展安全生产专项整治，严厉打击非法违法生产经营行为。不断加强社会治安综合治理，开展严打整治专项行动，“八类”刑事案件立案同比下降25%。加强公安队伍建设，涌现出陈首杰等一批先进典型。

　　——我们坚持务实办事，着力推进功能完善和品位提升，城市建管力度加大。以“规划先行、建管并重、经营城市”为重点，着力优化城市布局、不断加大投入力度、增强城市承载能力。积极推进城市总规修编，完善了煤矿棚户区改造、采煤沉陷区治理搬迁、城市道路升级改造、南出口景观带等专项规划。投资1.2亿元，新建、改建了南山路等13条道路和24条街巷路，对煤城路等60条道路进行维修养护。对12条道路和团结桥、奋斗桥、新世纪广场、迎宾门进行亮化升级，亮灯率达到98%以上。改造扩建了公路客运枢纽站和新街基步行街。投资1.4亿元，实施供热四期扩建工程，改造老旧供热管网28.6公里，新增集中供热7万平方米。市政净水厂升级改造工程和市区污水管网工程按计划完工。鹤翔新能源城市燃气管网和汽车加气站项目加快推进。开展了“全市治理违章建筑行动”，共拆除违章建筑397处，整治“弃管楼”1486栋。实施污染物减排工程33项，化学需氧量、氨氮、二氧化硫、氮氧化物等4项指标削减量完成国家和省下达的减排目标。淘汰“黄标车”及老旧车辆2704台、小锅炉55台。完成工程造林1.6万亩，义务植树84万株。开展PM2.5等6项指标的自动监测，空气质量达到国家二级标准。扎实推进农村生态建设，19个乡镇被命名为省级生态乡镇，3个乡镇晋升为国家级生态乡镇。用市场化运作的方式，改造了儿童公园娱乐设施，启动了城区地下弱电管网建设工程。

　　——我们坚持依法行事，着力推进职能转变和执政能力，自身建设得到加强。坚持把“目标导向、问题导向、底线思维”贯穿到政府自身建设始终，着力解决“不严不实”突出问题，持之以恒转作风、树新风、强政风，加快推进“四型”政府建设。推进了高效政府建设。按要求完成了政府机构改革，政府工作部门由35个精简到32个。开展了事业单位分类改革，分类批复事业单位264家。开展了“三单一图”改革，权力事项由3129项精简到2528项，梳理出责任事项1.8万余项，权力清单、责任清单正式向社会公布。推进了商事制度改革，将134项工商登记前置审批改为后置审批，强化事中事后监管。实施了“四证合一”登记制度，企业登记审批时限压缩了2/3以上。开展了行政事业性收费制度改革，公开了81个收费项目的标准和依据。推进了法治政府建设。自觉接受市人大法律监督、工作监督和市政协民主监督，主动听取各民主党派、工商联和社会各界意见。决策咨询和法律顾问作用全面增强，政府合同合法性审查和行政复议规范化不断提升。推进了诚信政府建设。扩大政务公开范围，升级市政府门户网站，市级财政预决算、“三公”经费支出、政府采购、公共资源交易、重大工程招投标等重点领域信息全部向社会公开。坚持以政府诚信引领社会诚信，努力营造诚信光荣、失信可耻的社会环境。推进了廉洁政府建设。认真落实 “一岗双责”，严格遵守廉洁从政有关规定，加强了对公共资金、国有资源、宏观政策措施落实、领导干部履行经济责任制情况的审计监督，认真执行中央和省、市委“八九十项”规定，开展办公用房清理、“吃空饷”治理，公务支出和经费管理步入规范化、制度化轨道。

　　统计、物价、外侨、老龄、双拥、人防、地震、气象、档案、民兵、预备役、关心下一代等工作取得新的进展，民族宗教、妇女儿童、残疾人、红十字等事业取得新的进步。

　　各位代表！面对特殊复杂的困难时期，这些成绩的取得实属来之不易。这是市委正确领导、科学决策的结果，是市人大、市政协有效监督、鼎力支持的结果，是历届班子薪火相传、求真务实的结果，是全市上下戮力同心、拼搏进取的结果。在此，我代表市政府，向全市人民，向各民主党派、工商联、无党派人士、人民团体和社会各界人士，向中省市直各单位，向驻军指战员、武警部队官兵和公安干警，表示衷心的感谢并致以崇高的敬意！

　　在肯定成绩的同时，我们也清醒地认识到，我市经济社会发展，还存在诸多不容回避的困难和问题。主要是：经济下行压力比预想的更加严峻复杂，煤炭、粮食等主导行业“量价齐跌”，对整体经济的影响持续加重；城市转型面临严重制约，结构性、资源型和体制性等深层次矛盾仍然突出；非公经济、新兴产业、中高端产品比重小，特别是工业结构单一，“一煤独大”；石墨等具有比较优势的矿产资源精深加工程度不够、附加值不高，资源优势尚未真正形成经济优势；财政收支压力有增无减，政府性债务进入还款高峰期，城市建设管理、公共服务和民生改善投入不足，与人民群众的期盼仍有较大差距；安全生产形势严峻，主体责任、领导责任、行业监管责任、行业管理责任和综合监管责任的落实亟需加强；支持龙煤鹤矿公司脱困发展压力较大；个别领导干部执行力不强，个别行政部门工作效率不高，个别公职人员不作为、乱作为等问题仍比较突出。对于上述问题，我们必须高度正视，马不停蹄地谋克难之策，一刻不停地思改进良方。

　　二、“十三五”时期奋斗目标和2016年主要工作

　　各位代表！从今年开始的未来五年，是我市实现经济解困、城市转型的关键时期。“十三五”时期，我市经济社会发展的主要预期目标是：地区生产总值年均增长11.4%；规模以上工业增加值年均增长33%；固定资产投资累计实现1100亿元；公共财政收入年均增长14.9%；社会消费品零售总额年均增长8%；外贸进出口总值年均增长5%；城镇居民人均可支配收入年均增长6.5%；农村居民人均可支配收入年均增长7%。总体安排上分两步走：前两年，打基础、增后劲，到2017年实现经济解困；后三年，抓提速、快发展，到2020年实现城市转型，经济社会步入健康可持续发展轨道，与全国、全省同步进入小康社会。

　　为实现上述目标，必须牢固树立五大发展理念，即树立创新发展的理念，把创新作为经济解困、城市转型的第一动力；树立协调发展的理念，把协调作为经济解困、城市转型的内在要求；树立绿色发展的理念，把绿色作为经济解困、城市转型的永恒主题；树立开放发展的理念，把开放作为经济解困、城市转型的必由之路；树立共享发展的理念，把共享作为经济解困、城市转型的根本目的。

　　今年是全面建成小康社会决胜阶段的开局之年。做好今年的工作，对于我市当前解困和长远发展，都具有重要意义。必须立足实际，准确研判形势，保持战略定力，增强发展信心。

必须清醒看到，鹤岗正处于迎难而进的攻坚期。从国际看，世界经济仍在深度调整，总体复苏疲弱态势难有明显改观。从国内看，我国已进入发展增速换挡、发展方式转变、发展动力转换、经济结构调整时期，经济下行压力还在加大。从全省看，煤矿城市仍面临困难的发展环境，煤炭供大于求形势难有大的改观，市场竞争将更加激烈。从我市看，经济总量不大、结构不优，质量不高，发展速度缓慢，部分行业和企业生产经营困难，经济发展面临的不确定、不稳定因素增多，对民生保障、城市建设、安全生产、社会稳定、财政运转等方方面面工作造成了巨大的压力，这种状况短期内难以扭转，实现经济解困、城市转型，我们面临的形势依然严峻，任务艰巨繁重。

　　必须清醒看到，鹤岗正处于抢抓机遇的关键期。国家新一轮支持东北地区老工业基地振兴，以及我省深入实施“五大规划”发展战略、建设“龙江丝路带”、落实煤炭城市转型发展规划，标志着我市迎来了全面振兴发展重大政策机遇；国家和省着力实施供给侧结构性改革，去产能、去库存、去杠杆、降成本、补短板，有利于我们加快技术进步、有效化解落后产能、提高经济增长质量和效益；国家和省重视资源型城市转型发展，关注铁路公路、农田水利、基础设施、健康养老等重点领域，有利于我们争取国家和省的项目和资金支持；国家和省不断深化财税、金融、国企等重点领域改革，有利于释放市场主体活力，对于三次产业发展、保障改善民生、龙煤鹤矿转制都将起到积极的促进作用。

　　必须清醒看到，鹤岗正处于稳定向好的发展期。经过近3年的深度调整、强筋固本、积攒后劲，支撑全市经济发展的利好因素不断集结，新的增长动能开始集聚发力，多点支撑、多极发展态势正在形成。通过两轮产业项目建设攻坚战，一些项目将逐步落地释放成产能，发力见效；规上企业整体发展势头看好，有望拉动今年经济增长；随着非煤产业新的增量，特别是石墨产业比较优势突出，发展前景广阔，将成为鹤岗转型腾飞新的主导力量；煤炭行业对整体经济的影响逐渐降低，全市工业形势有望好于去年；今年也是保障性安居工程建设投资最大的一年，将对经济增长产生综合、重要的拉动作用。各县区、各部门凝心聚力、和衷共济，全市上下风清气正、心顺劲足，经济解困的动能越来越足，城市转型的氛围越来越浓。

　　今年政府工作的总体要求是：认真贯彻落实党中央、国务院和省委、省政府的一系列重大决策部署，以“四个全面”战略布局为统领，牢固树立创新、协调、绿色、开放、共享的发展理念，以加快经济解困、城市转型为主线，坚持稳中求进工作总基调，全面落实“十三五”规划，坚持“调整结构、优化产业、大上项目、促进转型”发展思路不动摇，全力实施“延伸煤、做大墨、提升绿、繁荣贸、激活游”主体措施，促进“五大产业”加快发展，进一步培育新的发展动能，改造提升传统比较优势，增强持续增长动力，着力稳增长、促改革、调结构、惠民生，精准发力、扎实起步，为实现全面建成小康社会的宏伟目标奠定坚实基础。

今年全市经济社会发展的主要预期目标是：地区生产总值增长8.5%，规上工业增加值增长10%，固定资产投资增长12%，公共财政收入增长8.8%，社会消费品零售总额增长10%，进出口总值增长7%，城镇居民人均可支配收入、农村居民人均可支配收入增长7%和6.5%。为实现上述目标，将着力抓好以下8个方面工作。

　　（一）坚定不移地抓招商、上项目，进一步提升项目对经济解困、城市转型的牵动力

　　全方位招商引进项目。要把借外力作为全市解困转型发展的长期战略。紧盯发达地区、央企、大型民企尤其是500强企业抓招商，实现鹤岗经济在更大空间中的互融发展；紧盯长三角、珠三角、环渤海等产业转移地区抓招商，主动承接符合我市实际、有利产业发展的转移产能；紧盯成长性好、市场潜力大的科研成果抓招商，加快引进一批投资规模大、科技含量高、财税贡献多的质量效益型项目；紧盯优势资源和产业基础抓招商，有针对性地引进整合开发我市煤炭、石墨、绿色食品、生态旅游等产业资源的项目落地；紧盯项目生成抓招商，推进线索变意向、意向变协议、协议变合同、合同变落地，提高产业项目的生成率；紧盯创新方式抓招商，采取专题招商、产业链招商、会展招商、商会招商和以商招商等方式，力争实现招商工作取得新突破。

　　深层次推进落地项目。继续实施产业项目三年攻坚行动，推进省级重点项目18个、市级重点项目107个，总投资额200亿元以上，当年计划投资60亿元以上；实施大项目攻坚行动，筛选出全市投资5亿元以上大项目，单独确定任务重点推进；实施PPP项目库建设行动，以谋划城市基础设施项目为核心，加强项目库建设，重点推进项目前期工作；实施向上争取行动，以向上对接争取扶持政策为核心，围绕国家支持东北老工业基地振兴、“中国制造2025”和“全省制造业三年行动计划”等一系列产业政策，加大工作力度，切实把国家和省政策红利转化为项目落地。

　　多角度搭台承载项目。继续理顺市经开区工作体制机制，强化园区职能，提升规划、建设、管理、服务等综合功能。以“1区6园”为框架，完成总体规划和专项规划编制。重点抓好松鹤高新技术产业园、清源湖绿色食品产业园、鹤南煤化工产业园以及中小微企业创业园等园区的建设。探索政府主导、市场化运作、国有公司运营的发展模式，推动园区建设。通过国有公司的控制力、主导力和市场运营能力，完善园区路、电、水、通讯等基础设施和生产要素整合，推进项目向园区集中，产业向园区集聚。

　　（二）全力以赴地抓运行、调结构，进一步提升工业对经济解困、城市转型的支撑力

　　着力抓好工业运行。认真落实好中央和省、市促进工业经济稳增长的政策措施，充分发挥“双攻坚”行动10个推进组在项目建设、经济运行等方面的作用，坚决打好“规上工业保卫战”。要切实加强对煤炭开采和洗选、农副产品加工等重点企业的监测分析，帮助企业解决在市场、资金、原料、运输等方面存在的问题。推进地方煤矿整顿关闭和各类停产半停产企业开工，启动存量保运行。支持华能鹤电稳定发展，支持龙煤鹤矿脱困发展，支持中海油华鹤加快发展。

　　着力抓好结构调整。加快构建特色鲜明、结构优化、体系完整的立市产业群。一是依托主导产业调结构。做深做长煤炭精深加工业，把煤炭资源深度转化作为鹤岗经济解困、城市转型的基础，围绕煤转电、煤制焦、煤制气、煤制肥、煤基多联产等5大产业链，重点推进中海油华鹤50万吨复合肥、2×35万热电联产、征楠煤化工干熄焦、鹤翔新能源二期、中石化煤基多联产等项目。做大做强高端石墨产业，鹤岗未来发展“基础在煤炭，腾飞靠石墨”，要把发展石墨精深加工产业作为立市产业，以规划为引领，加大勘查力度，加快矿权整合，引进投资主体，拉长产业链条，壮大产业集群。重点推进建设“2个基地”，即高纯石墨生产基地和石墨烯生产基地；“5个产业链”，即新能源材料类石墨产业链、高档石墨密封类材料产业链、冶金耐火类石墨产业链、电碳石墨材料产业链和新兴材料产业链；“2个园区”，即鹤鸣石墨精深加工产业园和云山石墨综合开发产业园；“1个中心”，即鹤岗石墨新技术应用开发中心，把鹤岗打造成“空间布局合理、产业配套完善、产城有机融合”，中国一流、世界知名的“千亿级石墨产业城”。做精做深绿色食品产业，围绕水稻、玉米、大豆深加工和畜牧养殖、林产品加工等5大产业链，积极开发功能食品、休闲食品和保健食品。加大品牌营销力度，通过媒体宣传、展会宣传、市场宣传等综合手段，把我市知名的绿色食品品牌在全国打响，提升鹤岗品牌的影响力和竞争力。通过兼并重组、资本运作，整合域内粮食加工企业，从根本上扭转粮食加工企业面临的困境。重点推进万源粮油三期、吾朋公司大米蛋白粉二期、利农公司30万吨玉米深加工和绥滨50万只肉鹅加工等项目。二是扶强扶壮传统支柱产业调结构。充分发挥域内石灰石、粉煤灰、煤矸石、石墨尾矿等资源优势，改造提升建材产业；依托森工、国有林场和丰富的境内外林木资源，发挥骨干企业作用，发展高档实木家具、仿古家具、欧式家具，改造提升木材加工业；注重挖掘小兴安岭山野产品和地方土特产品的优势，重点推进兴汇公司万吨山葡萄酒、大兴公司南瓜饮品项目，加速项目生成，改造提升饮品制造业。三是加快发展新兴产业调结构。新材料产业，突出陶粒页岩综合开发利用，重点扶持鹏程科技公司年产500公里高铁轨道交通吸音板项目。生物与新医药产业，突出新药研发、中药和中成药的二次开发，重点扶持三精千鹤药业、未名药业和双兰星制药等企业，加快新产品开发，力争用2至3年时间，实现医药产业产值倍增。信息技术产业，突出发展系统软件、管理软件、网络安全软件和数据库，重点推进海雅科技服务外包、优天易云云计算等项目。新能源产业，突出自然资源利用、粮食深加工废弃物资源利用和工业余热余压资源利用，重点推进大唐风电二期、海润集团光伏电站一期、鑫塔水泥和成金建材余热发电项目。

　　着力抓好科技创新。深入实施创新驱动发展战略，积极构建科技创新体系，大力引进科研机构、大院名校共建创新载体，鼓励其在鹤岗设立技术转移中心、中试基地、试验中心和数据中心。大力支持企业研发机构建设，强化产学研用结合，加速科技成果和专利技术转化。今年，重点实施科技计划项目30项，新建研发机构 3个。坚持以市场为导向，支持企业开发新技术、新产品、新工艺，形成自主知识产权，推动产品升级换代，增强核心竞争力。年内专利申请量达到200件以上，新培育省级专利优势企业2户。继续实施“全省千户科技型企业三年行动计划”，大力引进科技人才、科技成果、科技项目和科技资金，力争培育科技型企业35户，确保实现三年100户的目标，形成新的增长动能。

　　（三）坚持不懈地抓三农、促增收，进一步提升农业对经济解困、城市转型的拉动力

　　发展现代农业。稳产能，继续推进亿亩生态高产标准农田建设，确保粮食总产稳定在28亿斤以上。调结构，着眼于市场需求变化和产业优势，把我市的优势作物调强、产业布局调优、经济效益调高。在巩固种植业结构调整成果的基础上，大力发展畜牧业，实施好“两牛一猪一禽”发展规划，建设一批标准化规模养殖场，实现“粮变肉”、“草变乳”过腹增值，今年畜牧业产值要增长10%以上。大力开展林下养殖、种植及营销，发展森林食品、森林畜禽、北药、食用菌生产、加工和营销。提品质，依托生态优势，持续推进农业“三减”，使更多农产品达到绿色、有机标准。今年，绿色食品基地认证面积要达到135万亩。增效益，推动农业由“种得好”向“卖得好”转变，进一步促进农业产业化发展。今年农业产业化龙头企业要达到52家。要进一步完善农产品市场营销体系，借助“互联网+农业”，拓宽线上线下销售渠道，让农民在扩大销售中增加收入。

　　推动农民创业。在思想发动、典型示范、“帮、学、干”结合基础上，充分调动农民创业的积极性，推广农民创业致富路径，引导农民从事能够增加收入的种植、养殖、销售、加工等多种创业活动。加大新型农民技能培训力度，提高农民就业能力。

　　建设美丽乡村。继续巩固农村环境卫生建设成果，打好环境卫生整治攻坚战，推进农村环境持续改善。按照美丽乡村建设标准、要求，全面推进“六改”工程，集中治理“五乱”现象，扎实开展“四化”工作，加快推进5个示范村和23个达标村建设，确保美丽乡村建设三年行动计划高标准、高质量完成。

　　推进精准扶贫。坚持精准识贫、精准扶贫、精准脱贫，加大扶贫开发力度，推进重点贫困村整村脱贫。因地制宜、因村施策、因户施法，积极向上争取生产扶贫、基础设施扶贫、公共服务扶贫等项目，绥滨县2017年底前必须脱贫摘帽。

　　深化农村改革。加快新型农业经营主体建设，新增农民专业合作社20个，千亩以上家庭农场、专业大户和从事农业生产企业分别新增20个。加快推进农村土地制度改革，全面完成农村土地确权工作，完善农村土地流转信息网络和服务平台建设。

　　（四）多措并举地抓市场、兴业态，进一步提升三产对经济解困、城市转型的贡献力

　　大力发展商贸流通业。积极引导现有商贸企业通过新业态、新模式植入改造，适应新常态，实现更高层次的发展。扶持引进城市综合体，以引导城市生活消费新潮流、新体验为方向，形成具有独特竞争力的新型商务模式。积极推进“互联网+流通”行动，推动传统商贸企业依托线下网点渠道资源、商品品牌和服务优势，自建线上平台或利用第三方平台发展电子商务，实现线上线下资源优势互补和协调应用。按照“一个中心、多点式”的发展格局，以工农、向阳主城区为中心，发展体验式购物中心、特色商业街等商业模式，为市民多层次消费提高平台。大力推进“便利消费进社区、便民服务进家庭”，形成“多点式”的社区商业中心。大力推进专业市场建设，重点推进浙江新农农产品批发大市场和建材大市场等项目。加紧构建农村现代商贸流通体系，鼓励开展“农批零对接”、“农超对接”、“农社对接”模式，向合约交易、订单交易、拍卖交易等现代化交易方式发展。

　　大力发展旅游业。以优良生态、特色文化、边域风情、绿色食品、精品景区为依托，推动旅游点、线、面合理布局，全面激活旅游业。启动实施鹤岗市旅游发展总体规划，围绕“一城、一带、四区”建设，重点打造以松鹤公园、清源湖为中心的鹤岗西部旅游集散区；以北方共青城为中心的大农业、大湿地、垦荒红色旅游集散区和以龙江三峡为中心的界江旅游集散区。整合域内旅游资源，推动三峡旅游资源投资运营公司快速发展。加大宣传推介力度，对接周边知名旅游景区，加强与国内百强旅行社合作，组建鹤岗旅游北京营销中心，精心推出界江风情游、冰雪休闲游、森林生态游、湿地观光游、近郊农业游等一批精品旅游线路。

　　大力发展文化产业。深度挖掘具有鹤岗印记、鹤岗符号、鹤岗特质的文化内涵，全力打造富有地域特色的城市文化。积极开发玛瑙、工艺保健药枕、传统立体仿古画等一批具有我市特色的民间艺术产品。加快推进金羽丝路创意产业基地、名山影视文化产业园建设，筹建艺术品交易中心。积极推进“文化+旅游”行动计划，拓展文化创意与旅游业的技术融合、产品融合、业务融合和市场融合，开展文艺团体驻旅游景区演出，实现文化创意与旅游业深度融合，提升文化产业的竞争力和影响力。

　　大力发展养老服务业。坚持以政府为主导，鼓励社会资本投入养老产业，探索公有民营、民建公助等机制，加强机构养老、社区养老和居家养老，办好公办保障性、民办公益性的养老服务机构。积极推进养老服务业与游、医、娱、餐、购、学“六结合”，今年重点推进建设2千张床位的养老机构2至3家、两百张床位的5至8家。运用“互联网+”等手段，积极培育一批居家养老服务企业和机构，为老年人提供助餐、助浴、助洁、助急、助医等规范化、个性化的家政服务。进一步拓宽养老服务产业领域，促进养老服务产业与文化、旅游、保险业良性互动发展。

　　大力推进对外经贸合作创新发展。以我省“东部陆海丝路经济带”建设为契机，发挥沿边优势，全方位推进对俄经贸的深度交流与合作。认真落实好我市与俄犹太州在多个领域达成的双边合作意向，重点在对俄矿产、林业、农业等方面，进一步加强合作。加快推进对外经贸转型升级，坚持以对俄资源开发、对韩国及台湾地区食品加工、对日本技术合作、对港澳地区资本引进为主攻方向，努力构建开放型经济发展新格局。深化“出口抓加工，进口抓落地”，大力抓好地产品出口加工和对外投资合作项目。加快发展和壮大对外经贸主体队伍，实施“外经贸主体壮大工程”，通过招商引资不断引进和培育大的外经贸主体，重点吸引两头在外的加工贸易企业和对俄战略投资者到我市落户经营，全年新增外经贸主体户数要达到20户以上。

　　大力发展现代金融业。鼓励金融机构充分运用现代金融工具，不断创新金融产品和融资模式，扩大资金投放规模，努力提高金融服务水平。继续实施“引金入鹤”工程，加快组建鹤岗市农商银行，支持发展融资性担保机构、小额贷款公司和融资租赁公司。改革政府融资模式，推进PPP模式，探索国有资产证券化，引导社会资本参与公共基础设施建设。加快中小微企业和农村信用体系建设，努力在社会营造“守信激励、失信惩戒”氛围。建立健全贷款保证保险制度，严格防范和处置非法集资，严禁各类投资公司、典当行和担保公司超范围经营，防止区域性金融风险发生。

　　（五）务实创新地抓建管、提功能，进一步提升城市对经济解困、城市转型的承载力

　　推进基础设施建设。加快铁路、公路、水路、空路“四路同建”。全力推进鹤佳快铁和园区铁路建设；启动鹤大公路南出口段、绥名公路、城市环路国防路段和林区旅游公路等改造扩建工程；加快名山港口码头改扩建，打开江海联运通道；加快鹤岗支线机场规划建设。完善城市路网，开工建设煤城路、兴建路等10条道路，新改扩建30条街巷道路。加大“一路、一带、一河、一城”建设改造力度，彻底解决路窄、河脏、房破、景差的问题，打通哈肇公路与鹤大公路的连接，实现“南出口”车辆分流，缓解城市主干道交通运输压力。加快水、电、热、气“四网同建”。新建、改造农村饮水安全工程10处，再造及延伸管网13.3公里；完成关门嘴子水库可研报告批复，力争形象开工。深入实施城网、农网改造工程，重点推进鹤岗500千伏输变电工程。继续推进供热四期和全市供热老旧管网改造。加快燃气网建设，重点推进鹤翔新能源城市燃气管网和汽车加气站项目，改造部分燃气老旧管网。

　　推进城市综合管理。树立“大城管”理念，进一步加强城市综合执法，推进网格化、数字化和精细化城市管理。加大环境整治力度，不断提高环卫保洁质量标准和管理水平，加速环卫工作全面提档升级。实施路灯节能改造工程，继续加大城区亮化力度。加大违法用地、违法建筑的治理力度，健全完善“两违”监管机制和查处机制。加强“弃管楼”社区物业服务站建设，实现老旧小区物业基本覆盖。实施城市园林绿地改造提升工程，进一步完善公园、广场设施。强化“三供两治”的管理力度，保障相关设施安全运行。积极谋划推进海绵城市和地下综合管廊建设，开展黑臭水体治理改造。启动建设大型公共停车场。

　　推进生态环境保护。开展节能减排，强化污染防治，严格执行新建项目“三同时”制度。抓好秸秆禁烧、淘汰“黄标车”和老旧车辆及燃煤小锅炉治理。推进生活垃圾中转站建设。落实国家和省大气、水污染防治行动计划，实施域内大气、水体达标治理工程。完成全民义务植树60万株，植树造林1.5万亩。推进国家和省级生态县、生态乡镇及生态村创建活动。

　　（六）持之以恒地抓民生、促和谐，进一步提升群众对经济解困、城市转型的向心力

　　努力扩大创业就业。实施更加积极的创业就业政策，认真落实小额担保贷款和灵活就业人员社保补贴政策。加强公共就业服务，搞好职业技能培训，提高创业就业成功率。实现新增就业2.1万人，城镇登记失业率控制在4.1%以内。大力推进大众创业、万众创新，着力培育发展壮大非公经济。要拓宽发展领域，继续清理各种形式的不合理规定，消除各种隐性壁垒，主动对接国家600亿中小企业发展基金，落实用地、财税、金融等扶持政策。通过积极挖掘市场机会、放开准入领域、公平参与公共资源项目竞争，培育和扶持中小微企业发展壮大，促进非公经济持续健康发展。

　　切实加强社会保障。加快完善社会保障制度，全面开展社会保险全民参保登记工作，整合城镇居民基本医疗保险和新型农村合作医疗保险两项制度，建立统一的城乡居民基本医疗保险制度，进一步提高基本医疗保险政策待遇水平，落实失业保险稳岗补贴政策。进一步扩大临时救助范围，做好各类困难群众救助工作。

　　加快保障性安居工程建设。城市棚改新建3106套，续建7628套；煤矿棚改新建8306套，续建5439套；公共租赁住房续建1861套；改造城中村7742户；改造重点城镇棚户区933户；改造采沉区棚户区10000户。

　　大力发展社会事业。加大教育投入，整合教育资源，全面提升学前教育、义务教育、高中教育、职业教育、高等教育和特殊教育水平。加快学校标准化和信息化建设步伐，完成3个市辖区的基本均衡县区建设任务；创建省级改革发展示范校2所。加快职业教育资源整合，优化职教布局，发展现代职业教育。加快医疗卫生事业发展，开展健康龙江行动，大力发展健康服务业，抓好市中医院“医养康”综合体二期项目建设，构建医养康健康服务新模式。深入开展“医疗服务质量提升年”活动，实施社区卫生服务提升工程，为群众提供方便、快捷、优质、高效的医疗卫生服务。实施全面二孩政策。大力发展文化事业，着力完善城乡各级公共文化基础设施，加快市县两级图书馆、艺术馆和文化馆达标建设，推进社区文化中心、文化室建设，建成广播电视网站，实现传统媒体与新媒体的融合。积极发展体育事业，广泛开展全民健身活动，加快群众身边的体育场地设施建设，水上健身中心主体完工，城市社区体育场地设施覆盖率达到30%。不断提高竞技体育水平，积极备战全国第十三届冬运会。切实加强人才工作，健全人才工作机制，加大人才引进力度，加强领军人才梯队建设，完善人才创业创新基地，为转型发展提供人才保障。

　　（七）毫不懈怠地抓安全、保稳定，进一步提升社会治理对经济解困、城市转型的保障力

　　抓好安全生产工作。认真履行“党政同责、一岗双责、失职追责”和“三个必须”要求，全面落实安全生产主体责任、领导责任、行业监管责任、行业管理责任和综合监管责任，加强各级安全生产监管机构和执法队伍建设，配齐配强各级安全监管人员。切实强化煤矿安全生产管理。加强煤矿“一通三防”工作，强化水、火、瓦斯、粉尘等灾害治理，以标准化建设促进安全生产工作。严格煤矿安全监管，从严打击违法违规行为。进一步推进地方煤矿整治整合关闭工作。切实抓好非煤矿山、特种设备、危险化学品、烟花爆竹等行业安全监管，加强道路交通、消防森防、食品药品、建筑施工等重点领域和商场、学校、养老机构、幼儿园等人员密集场所的安全生产工作。严格执行安全生产一票否决和责任追究制，从严查处各类违法违纪人员，坚决用最严格的监管、最严厉的处罚、最严肃的问责，最大限度地减少一般事故、控制较大事故、防范和遏制重特大事故的发生。

　　抓好信访稳定工作。认真落实信访工作责任制、信访接待日和重点案件领导包案制度，积极化解信访积案，切实解决好群众反映强烈的问题。坚持依法治访，完善人民调解、司法调解和行政调解联动机制，建立畅通、规范、有序的群众诉求表达、利益协调和权益保障渠道。按照法定途径分类处理信访投诉请求事项，妥善化解社会矛盾，确保群众提出的投诉请求依法依规解决。

抓好社会综治工作。深入推进“平安鹤岗”建设，完善立体化社会治安综合防控体系，加大公安基层基础和科技建设的力度，科学有效地预防和处理突发性群体事件，强化对特殊人群和虚拟社会的管理服务，高压打击各类违法犯罪活动。进一步加强各级政府的应急指挥平台建设，完善应急管理机制，有效应对各类突发事件，确保社会和谐稳定。

　　（八）一以贯之地抓深化、解难题，进一步提升改革对经济解困、城市转型的支持力

　　深化行政审批制度改革。做好已取消和下放管理层级行政审批事项的落实和衔接。动态完善权力清单、责任清单和中介服务清单，推动建立负面清单。继续清理和规范中介、协会、事业单位等具有的实质性审批权。推进中介机构、行业协会商会与主管部门、行政机关脱钩。全面推进不动产统一登记工作。着力构建以信用监管为核心的事中事后监管机制。

　　深化公共资源配置市场化改革。对集中供热热源、供气、供水、污水垃圾处理、医疗、教育、城市基础设施、公共网络、公共交通等所有经营性公共资源，都要采取招投标的方式进行公开配置。完善交易平台，提高资源配置的公平性和透明度。

　　深化财税、文教、卫生等重点领域改革。深入推进财政预算管理改革。实行预决算公开，全面实施预算绩效管理；加强政府债务管理，严格规范债务借、用行为，防范债务风险；清理税费优惠，规范财政税收优惠政策；全面强化税收征管和非税征缴；完善支出标准，改革支出方式，建立健全政府投资管理体系。加快文教卫生体制改革。健全中小学教师、校长定期交流制度，改革中考考试内容和高中招生办法，统筹义务教育资源均衡配置，逐步缩小城乡、校际差距。全面深化医药卫生体制改革，突出抓好县级公立医院改革，加强医联体建设，推进分级诊疗、双向转诊等制度建设，推动优质卫生资源向基层下沉。深化综合执法体制改革，推进重点领域联合执法。深化收费体制改革，取消、合并一批行政事业性收费项目，推行“一口制、一次性”收费。继续开展清人员、清资产、清资源、清资金的“四清”工作。

　　各位代表！做好今年各项工作，必须进一步加强政府自身建设。要巩固和扩大“三严三实”专题教育成果，坚持目标导向和问题导向相结合，坚持底线思维与法治思维相统一，努力建设人民满意政府。一要努力建设思想解放、创新发展的政府。主动适应新常态、新政策、新要求，坚决摒弃老思维、老套路、老办法。加快建设一支具有专业思维、专业素养和专业方法的干部队伍，提高政府工作专业化水平。牢固树立“五大发展理念”，转变不适应市场经济的思想观念。对不适应、不适合甚至违背新的发展理念的认识要立即调整、行为要坚决纠正、做法要彻底摒弃。二要努力建设依法行政、公开透明的政府。坚持把政府工作全面纳入法治轨道，运用法治思维和法治方式深化改革、推动发展、化解矛盾、维护稳定。坚持法定职责必须为、法无授权不可为，严格按照法定权限和程序行使权力、履行职责。依法接受人大及其常委会法律和工作监督，主动接受政协民主监督，严格执行人大及其常委会的决议决定，认真办理人大代表建议和政协委员提案，广泛听取民主党派、工商联、无党派人士、人民团体意见，自觉接受群众监督和舆论监督。深入推进政务公开，推进决策公开、执行公开、管理公开、服务公开和结果公开，让权力在阳光下运行。三要努力建设担当有为、务实重干的政府。摆脱换挡焦虑、克服本领恐慌，突破守摊子、保位子的观念束缚，敢抓敢管、敢闯敢干，少说多做、说到做到，真抓实干、勇争一流。以“功成不必在我”的思想境界和夙夜在公、尽心竭力的责任担当，为鹤岗经济社会发展汇聚强大的正能量。四要努力建设作风优良、服务高效的政府。持续改进工作作风，大力提升服务水平，紧盯发展目标任务，围绕招商引资、项目建设和企业发展，主动作为、优质服务，破解体制机制障碍，提高行政效率效能，不懈怠、不松劲，久久为功、善作善成，用作风大转变、服务大提升，助推经济解困、城市转型。五要努力建设廉洁自律、风清气正的政府。认真落实党风廉政建设“一岗双责”，坚决执行《中国共产党廉洁自律准则》和《中国共产党纪律处分条例》，深入开展反腐败斗争，严肃执纪问责，有腐必惩，有贪必肃。坚持用制度管权、管事、管人、管钱，扎紧制度笼子，防范权力越线。严格执行中央和省、市委“八九十项”规定，坚持倡俭治奢，推进公务用车制度改革，确保“三公”经费只减不增，集中财力保民生、促发展。

　　各位代表！新的一年开启新的希望，新的征程承载新的梦想。实现经济解困、城市转型的光荣使命，要求我们砥砺前行；全市人民的信任期盼，鞭策我们勇往直前。让我们在省委、省政府和市委的正确领导下，全面落实中央和省、市委的决策部署，倍加珍惜来之不易的发展成果，倍加珍惜十分难得的发展机遇，倍加珍惜社会和谐稳定的大好局面，团结带领全市人民，主动迎接挑战，积极奋发有为，坚持苦干实干，激活能量智慧，为加快实现经济解困、城市转型的目标，为鹤岗人民的富裕幸福而努力奋斗！
